# High-Resolution MR Characterization of Myocardial Infarction using Compressed Sensing with Edge Preservation

**DOI:** 10.1186/1532-429X-18-S1-P303

**Published:** 2016-01-27

**Authors:** Li Zhang, Jennifer Barry, Mihaela Pop, Graham A Wright

**Affiliations:** 1 grid.17063.33Medical Biophysics, University of Toronto, Toronto, ON Canada; 2 grid.17063.33Sunnybrook Research Institute, Toronto, ON Canada

## Background

Characterization of infarct heterogeneity can inform therapeutic strategies for ventricular tachycardia and the management of patients with prior myocardial infarction (MI)^1,2^. Multi-contrast late-enhancement (MCLE)^3^ images along the signal-relaxation curve, acquired in a breath-hold ECG-gated scan, offer better visualization of MI than conventional IR-FGRE. However, current MR images either with IR-FGRE or MCLE provide an inferior spatial resolution of 1.6-2.0 mm in-plane with a slice thickness of 5-8 mm in the clinical setting, which is not sufficient to properly delineate infarct heterogeneity. This work presents a three-dimensional MCLE sequence accelerated using variable density Poisson-disk sampling and also proposes a novel reconstruction method using Compressed Sensing with Edge Preservation (COSEP), achieving an *in-vivo* isotropic resolution of 1.5 mm within a single breath-hold.

## Methods

Three Yorkshire pigs with six-week-old infarcts were imaged after injection of Gadolinium-DTPA of 0.2 mmol/kg using 3D MCLE with a 160 × 160 × 10 acquisition matrix over a 1.5 cm-thick slab with corresponding resolution of 1.5 mm^3^. The undersampled datasets were prospectively acquired using Variable density Poisson-disk sampling patterns at a net acceleration of 5 with a 16-channel anterior cardiac coil array in a GE 3T scanner. The temporal signal-relaxation characteristic vectors were extracted from a training set, generated with a pre-defined model, using principal component analysis and then utilized to recursively transform spatiotemporal signal vectors to spatial principal component (PC) maps during the reconstruction. Local weighted total variation regularization^4^ was then applied to the CS framework to preserve anatomical edges in the infarct regions on the reconstructed spatial PC coefficient maps. For comparison, an alternative reconstruction method REPCOM^5^ was implemented and the IR-FGRE images were also acquired with the parameters as follows: matrix = 160 × 160, FOV = 24 cm and slice thickness = 5 mm.

## Results

**Figure 1:**
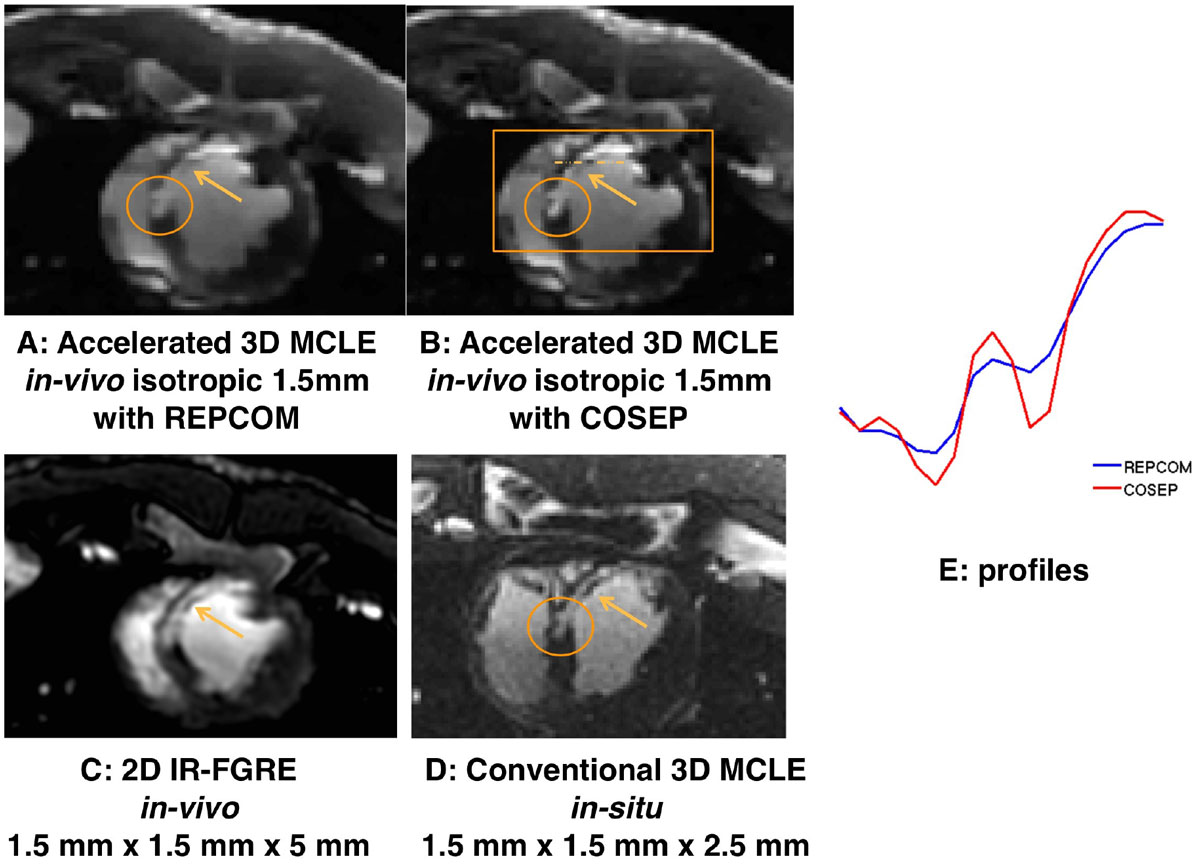
**the reconstructed magnitude images of a representative slice in the short axis view of an** ***in-vivo*** **pig heart at an inversion time of 180 ms**. A and B are reconstructed results from REPCOM and COSEP respectively. The bright rims along the wall of left ventricle indicated the infarct region. For COSEP, weighted total variation is locally applied to the area indicated by the box on B. C is the IR-FGRE image that corresponds to the representative slice. E shows two profiles from both REPCOM and COSEP, plotted across the dashed lines indicated in B. Following the in-vivo study, the pig was sacrificed 10 minutes after another injection of Gadolinium-DTPA of 0.1 mmol/kg and was then scanned using conventional 3D MCLE with an isotropic resolution of 1.5 mm. D shows the image corresponding to the representative slice acquired using conventional 3D MCLE in the *in-situ* study.

From A to D in Figure 1, COSEP provides the highest reconstructed spatial resolution on the magnitude image, computed exclusively within the area indicated by the box; the anatomical edges in the infarct region indicated by the arrow are well-preserved on the reconstructed image by COSEP; the reconstructed infarct branches within the ellipse shown by COSEP are more consistent with those on the conventional high-resolution 3D MCLE image acquired after sacrifice. In comparison with REPCOM in Figure 1E, COSEP presents sharper contrast between infarct and healthy myocardium along the signal intensity profile taken along the dashed line in Figure 1B.

## Conclusions

We successfully demonstrated that high-resolution characterization of myocardial infarction *in vivo* is feasible using accelerated 3D MCLE with the COSEP reconstruction. We have shown that an isotropic resolution of 1.5 mm was achieved within single breath-hold in an *in-vivo* prospective pig study.

